# New Methods, Old Brains—A Systematic Review on the Effects of tDCS on the Cognition of Elderly People

**DOI:** 10.3389/fnhum.2021.730134

**Published:** 2021-10-27

**Authors:** Anna Siegert, Lukas Diedrich, Andrea Antal

**Affiliations:** Department of Neurology, University Medical Center Göttingen, Göttingen, Germany

**Keywords:** transcranial direct current stimulation (tDCS), cognition, episodic memory, declarative memory, aging, elderly

## Abstract

The world's population is aging. With this comes an increase in the prevalence of age-associated diseases, which amplifies the need for novel treatments to counteract cognitive decline in the elderly. One of the recently discussed non-pharmacological approaches is transcranial direct current stimulation (tDCS). TDCS delivers weak electric currents to the brain, thereby modulating cortical excitability and activity. Recent evidence suggests that tDCS, mainly with anodal currents, can be a powerful means to non-invasively enhance cognitive functions in elderly people with age-related cognitive decline. Here, we screened a recently developed tDCS database (http://tdcsdatabase.com) that is an open access source of published tDCS papers and reviewed 16 studies that applied tDCS to healthy older subjects or patients suffering from Alzheimer's Disease or pre-stages. Evaluating potential changes in cognitive abilities we focus on declarative and working memory. Aiming for more standardized protocols, repeated tDCS applications (2 mA, 30 min) over the left dorso-lateral prefrontal cortex (LDLPFC) of elderly people seem to be one of the most efficient non-invasive brain stimulation (NIBS) approaches to slow progressive cognitive deterioration. However, inter-subject variability and brain state differences in health and disease restrict the possibility to generalize stimulation methodology and increase the necessity of personalized protocol adjustment by means of improved neuroimaging techniques and electrical field modeling.

## Introduction

The prognoses are alarming: by 2050 about 16% of the world's population will be aged over 65 (United Nations, 2019). With this comes a dramatic increase in the prevalence of age-related cognitive deterioration: in 30 years ~152 million people will be suffering from dementia, 60–70% of which with Alzheimer's Disease (AD) (World Health Organization, 2020). Although the body of research on neurodegenerative diseases is extensive, there is no intervention available to cure or to stop the progression of neurodegeneration and thus cognitive decline. This makes clear the necessity for novel treatment.

One of the recently discussed interventions among the novel treatment options is non-invasive brain stimulation (NIBS). The most common electrical stimulation method in the NIBS family used on humans is transcranial direct current stimulation (tDCS). Therefore, in this review we will focus on tDCS and its potential to interfere with age-related cognitive decline.

During tDCS constant weak electric currents (usually 1–2 mA) are applied to the cerebral cortex via external non-invasive electrodes to modulate neuronal excitability, firing rates and thus overall cortical activity (Priori et al., 1998; Nitsche and Paulus, 2000, 2001). Excitability changes are based on altered neuronal membrane potentials resulting in higher probabilities for de- or hyperpolarization (Purpura and McMurtry, 1965; Nitsche et al., 2003a; Lefaucheur et al., 2017). Depending on the direction of current flow (relative to orientations of neuronal axes) membrane potentials increase or decrease–with anodal tDCS being more likely to potentiate depolarization by increasing excitability, whereas cathodal tDCS tends to shift potentials toward hyperpolarization (Bindman et al., 1962; Purpura and McMurtry, 1965; Gorman, 1966; Nitsche and Paulus, 2000, 2001). However, these polarity-dependent predispositions cannot be generalized. Variations in several factors such as stimulation intensity (Batsikadze et al., 2013), duration (Nitsche et al., 2008; Batsikadze et al., 2013) or neuron orientation (more precisely somato-dendritic axis orientation) with respect to current flow (Kabakov et al., 2012; Rahman et al., 2013) may reverse excitatory into inhibitory effects and vice versa (Lefaucheur et al., 2017). Effects of tDCS have not only been observed online (during stimulation) but also offline (after stimulation) (Nitsche and Paulus, 2000, 2001; Nitsche et al., 2003c). Evidence from pharmacological studies suggests that tDCS impacts neuronal plasticity by modulating synaptic transmission via NMDA receptors (Liebetanz et al., 2002; Nitsche et al., 2003a, 2004) and GABA levels (Stagg et al., 2009). On a larger scale tDCS seems to affect functional network connectivity and the synchronization of neuronal populations across the cerebral cortex and within subcortical areas (Keeser et al., 2011; Polanía et al., 2011a,b, 2012).

In the past few years, based on the potential of tDCS to impact neuronal plasticity as well as network connectivity, tDCS studies have been extended to precisely investigate cognitive effects [for review see Shin et al. (2015)]. Evidence has been found that tDCS can modulate memory functions and enhance cognition in physiological (Berryhill and Jones, 2012; Hsu et al., 2015; Prehn and Flöel, 2015) as well as pathological aging (Flöel, 2014). Functional neuroplastic network modifications (Nitsche et al., 2003b) may compensate for age- and neurodegeneration-related cognitive impairments. Further, on the molecular level, tDCS may modulate or induce synaptic plasticity, which potentially results in longer-lasting altered learning and memory capabilities as long-term potentiation (LTP) and –depression (LTD) are thought to be the physiological basis of learning and memory (Bear and Malenka, 1994; Baudry, 2001; Braunewell and Manahan-Vaughan, 2001). Consequently, applying tDCS in the context of age-related cognitive decline [for review see Coffman et al. (2014)] seems promising to restore memory and prevent further deterioration.

TDCS treatment approaches, mainly using anodal stimulation, that can interfere with cognitive decline in early disease-stages appear particularly promising to prevent or slow disease progression such as in mild cognitive impairment (MCI) (Petersen and Negash, 2008). However, since re-discovery of tDCS ~20 years ago, scientists have applied electrical stimulation in multiple fashions varying montage, current intensity and polarization, and duration as well as the context of application (Lefaucheur et al., 2017). Therefore, tDCS experiments have revealed promising albeit highly variable effects on cognition (Elder and Taylor, 2014). Reining in the high variance through method standardization would be a necessary next step toward developing efficient treatment approaches.

Here we review the potential of tDCS to modulate cognitive functions in the elderly using the tDCS database (http://tdcsdatabase.com). The tDCS database is an open-access community-driven database that has been introduced to the scientific community by prestigious scientists of the field in 2018 (Grossman et al., 2018) and comprises 4.747 entries as of the writing of this review. It compiles mainly human tDCS studies that have been peer-reviewed and include all essential details on the application procedure as well as stimulation parameters (Grossman et al., 2018). Grossman et al. thereby aim to transparently provide scientists with all necessary information to develop efficient tDCS protocols and promote or improve clinical applications, facilitate meta-analysis across studies, and finally reduce variability of tDCS outcomes by optimizing experimental parameters based on previous evidence. For further details of inclusion criteria and maintenance of the database see Grossman et al. (2018).

We aimed to provide a comprehensive overview and further propose suitable tDCS procedures and parameters for future studies aiming to counteract cognitive age-associated deterioration. We focused on studies that investigated modulatory effects of tDCS to intervene with declarative and working memory deterioration as this is one of the major features of age-related cognitive decline (Rönnlund et al., 2005) and is accelerated in dementia (Reitz and Mayeux, 2014).

## Methods

### TDCS Database Research

**Figure 1:**
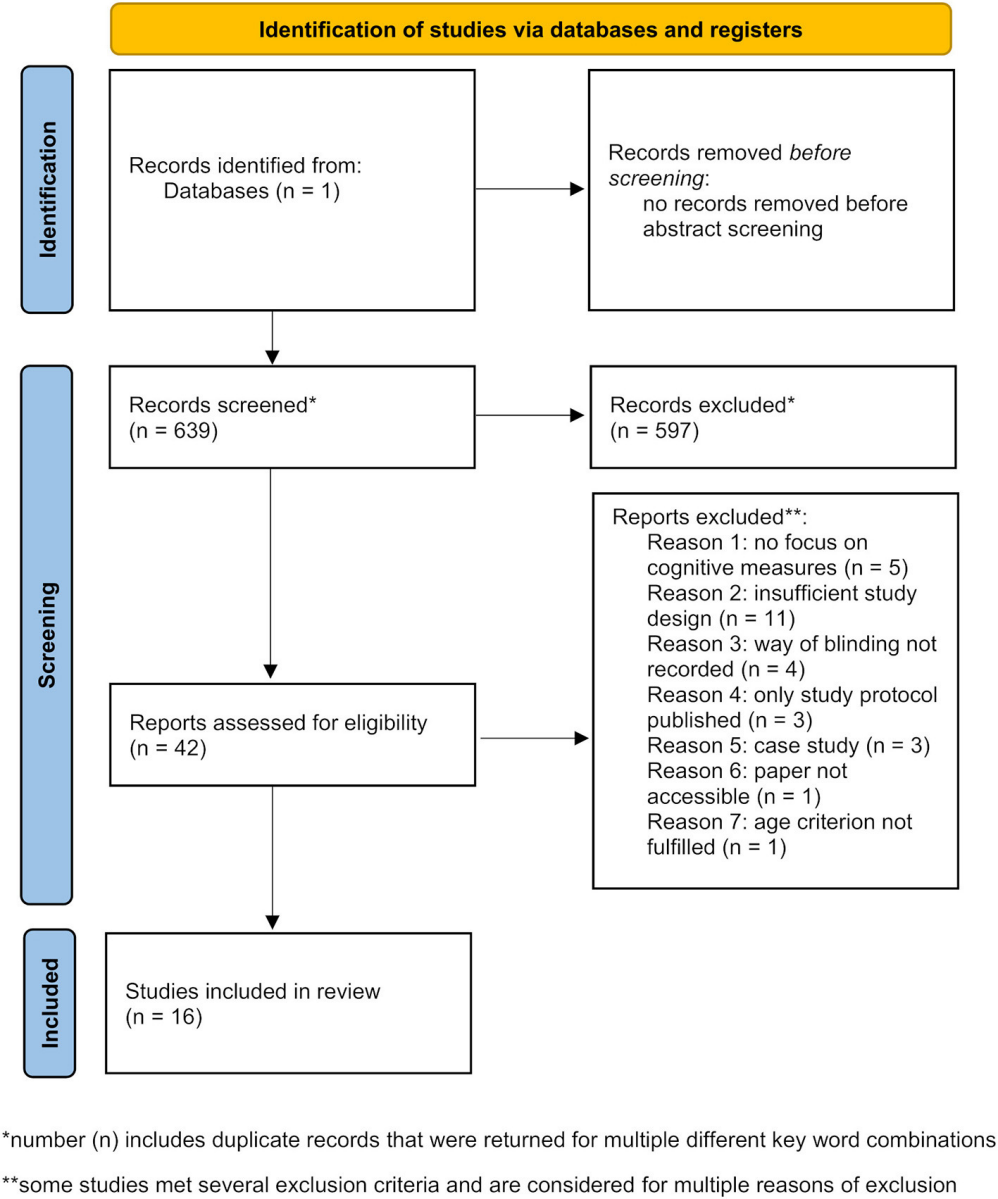
PRISMA flow diagram depicting identification, screening, and inclusion strategies for the selection of the reviewed studies [modified from Page et al. (2021)].

Literature database research was carried out in the tDCS database (http://tdcsdatabase.com) in February 2021. To ensure an efficient database screening several inclusion and exclusion criteria were determined. Inclusion criteria comprised: original paper on tDCS application(s) (previously unpublished data); subject age range starting ≥50 years (studies with old and young subjects were included if the old subject's age range started ≥50 years); focus on cognitive outcome measures of declarative or working memory and a double-blinded, randomized, and sham/placebo-controlled study design (unless it was a pilot or preliminary study). Aging is considered the strongest risk factor for MCI and AD. The prevalence of MCI is increasing dramatically wit age starting from 6.7% for individuals in the range of 60-64 years up to 25.2% for people in the range of 80–84 years (Petersen et al., 2018). A similar situation applies for AD with the first symptoms usually occurring after the age of 60 years (Ballard et al., 2011). With our age range starting ≥50 years we include all potential patients in early and later stages of disease. In this analysis we excluded reviews as well as meta-analyses, single-blinded or uncontrolled studies, case reports, and studies in which the blinding procedure was not mentioned or insufficiently described so that it could not clearly be extracted whether double-blinding was assured. The whole process of study identification, screening, eligibility assessment and inclusion was summarized in a PRISMA flow diagram (Figure 1).

### Keyword Search

Several keyword combinations were used to collect studies (that were further filtered according to above listed inclusion and exclusion criteria). Before precise filtering, abstracts were screened and all preliminary screening results were listed (Table 1). The following documentation of keyword search corresponds to the screening order whereby already included publications were not mentioned or listed again if repeatedly returned for other keyword combinations. To begin with, the keywords “transcranial direct current stimulation” or “tDCS” and “elderly” returned two studies that were directly excluded. Next, the search for “transcranial direct current stimulation” and “aging” revealed 186 studies. Abstract screening resulted in 12 studies considered relevant. Furthermore, “tDCS” and “aging” returned 24 additional studies of which two were selected. The keywords “transcranial direct current stimulation” or “tDCS” and “older” or “old” filtered out five studies of which 1 passed the abstract screening. Subsequently, the screening process was further specified. A combination of “transcranial direct current stimulation,” “cognition” and “aging” returned 19 studies with 1 relevant publication. Keyword filtering for “transcranial direct current stimulation” or “tDCS,” “memory” and “aging” added 1 more relevant publication out of 25 results, while “tDCS” and “memory” returned 34 studies of which five were considered relevant according to their abstracts. Another more focused search for “transcranial direct current stimulation” or “tDCS,” “memory” and “aged” resulted in 44 and for “transcranial direct current stimulation,” “cognition” and “aged” in 48 studies of which a total number of eight studies remained after abstract selection. Two more studies were found and included as they were cited in a review or one of the previously included papers. Finally, the keywords “transcranial direct current stimulation” and “memory” only returned 1 more relevant study out of 194 results as other appropriate papers were already included. Based on further evidence for relevant studies extracted from previous inclusions an author search for “Manenti” and “Sandrini” (four results), “Ferrucci” (41 results) and “Berryhill” and “Jones” (nine results) returned five other relevant studies. These were not found previously as they did not contain the keywords “transcranial direct current stimulation.” Four more recently published studies that seemed relevant were only available on PubMed but will subsequently be added to the tDCS database upon approval. The abstract screening eventually resulted in a list of 42 publications (Table 1) that were precisely filtered according to exclusion and inclusion criteria so that 16 studies remained to be reviewed (Table 2).

**Table 1:** List of all studies that passed the keyword and abstract screening in the tDCS database.

**Keywords (or other search key)**	**# Studies**	**Selected studies (PMID)**	**Title**	**References**	**Country**	Consideration for review^*^
Transcranial direct current stimulation, aging	186	31196835	Effects of 6-month at-home transcranial direct current stimulation on cognition and cerebral glucose metabolism in Alzheimer's disease	Im et al., 2019	South Korea	included
		33160420	Cognitive training and brain stimulation in prodromal Alzheimer's disease (AD-Stim)-study protocol for a double-blind randomized controlled phase IIb (monocenter) trial	Thams et al., 2020	Germany	included
		26923418	Older adults get episodic memory boosting from non-invasive stimulation of prefrontal cortex during learning	Sandrini et al., 2016	Italy	included
		26200716	Better together: Left and right hemisphere engagement to reduce age-related memory loss	Brambilla et al., 2015	Italy	excluded, (1) and (2)
		25449530	Transcranial direct current stimulation in mild cognitive impairment: Behavioral effects and neural mechanisms	Meinzer et al., 2015	Germany	included
		29050849	Neuronal and behavioral effects of multi-day brain stimulation and memory training	Antonenko et al., 2018	Germany	excluded, (3)
		28946572	Anodal transcranial direct current stimulation over the right hemisphere improves auditory comprehension in a case of dementia	Costa et al., 2017	Italy	excluded, (5)
		28707568	Effects of transcranial direct current stimulation on neural networks in young and older adults	Martin et al., 2017	Germany	excluded, (1) and (3)
		28314813	tDCS-induced modulation of GABA levels and resting-state functional connectivity in older adults	Antonenko et al., 2017	Germany	excluded, (1)
		27903289	Changes in cerebral glucose metabolism after 3 weeks of non-invasive electrical stimulation of mild cognitive impairment patients	Yun et al., 2016	South Korea	included
		27381076	Brain stimulation during an afternoon nap boosts slow oscillatory activity and memory consolidation in older adults	Ladenbauer et al., 2016	Germany	excluded, (2)
		27178247	Older adults improve on everyday tasks after working memory training and neurostimulation	Stephens and Berryhill, 2016	USA	excluded, (2)
tDCS, aging	24	24062685	Enhancing verbal episodic memory in older and young subjects after non-invasive brain stimulation	Manenti et al., 2013	Italy	excluded, (2)
		26696882	No significant effect of prefrontal tDCS on working memory performance in older adults	Nilsson et al., 2015	Sweden	excluded, (2)
Transcranial direct current stimulation (or tDCS), older	5	27247261	Boosting slow oscillatory activity using tDCS during early nocturnal slow wave sleep does not improve memory consolidation in healthy older adults	Paßmann et al., 2016	Germany	excluded, (2)
Transcranial direct current stimulation, cognition, aging	19	28062255	Differential effects of bihemispheric and unihemispheric transcranial direct current stimulation in young and elderly adults in verbal learning	Fiori et al., 2017	Italy	included
Transcranial direct current stimulation (or tDCS), memory, aging	25	26116933	Memory improvement via slow-oscillatory stimulation during sleep in older adults	Westerberg et al., 2015	USA	included
tDCS, memory	34	24678298	Anodal tDCS during face-name associations memory training in Alzheimer's patients	Cotelli et al., 2014	Italy	excluded, (4)
		22016735	Improved proper name recall in aging after electrical stimulation of the anterior temporal lobes	Ross et al., 2011	USA	excluded, (3)
		28485663	Can 8 months of daily tDCS application slow the cognitive decline in Alzheimer's disease? A case study	Bystad et al., 2017	Norway	excluded, (5)
		28509625	Direct-current stimulation does little to improve the outcome of working memory training in older adults	Nilsson et al., 2017	Sweden	excluded, (3)
		26250473	Effects of transcranial direct current stimulation upon attention and visuoperceptual function in Lewy body dementia: A preliminary study	Elder et al., 2016	UK	excluded, (1)
Transcranial direct current stimulation (or tDCS), memory, aged	44	28934620	Clinical utility and tolerability of transcranial direct current stimulation in mild cognitive impairment	Murugaraja et al., 2017	India	excluded, (2)
		28637840	Promoting sleep oscillations and their functional coupling by transcranial stimulation enhances memory consolidation in mild cognitive impairment	Ladenbauer et al., 2017	Germany	excluded, (2)
		27653887	At-home tDCS of the left dorsolateral prefrontal cortex improves visual short-term memory in mild vascular dementia	André et al., 2016	Germany	excluded, (1)
		27005937	Transcranial direct current stimulation as a memory enhancer in patients with Alzheimer's disease: A randomized, placebo-controlled trial	Bystad et al., 2016	Norway	included
		31529691	Randomized controlled trial of tDCS on cognition in 201 seniors with mild neurocognitive disorder	Lu et al., 2019	Hong Kong	included
		26499250	Would transcranial direct current stimulation (tDCS) enhance the effects of working memory training in older adults with mild neurocognitive disorder due to Alzheimer's disease: Study protocol for a randomized controlled trial	Cheng et al., 2015	Hong Kong	excluded, (4); actual study: PMID 31529691
		28390970	Transcranial direct current stimulation can enhance working memory in Huntington's disease	Eddy et al., 2017	UK	excluded, (7)
Transcranial direct current stimulation, cognition, aged	48	25379604	Transcranial direct current stimulation and cognitive training in the rehabilitation of Alzheimer's disease: A case study	Penolazzi et al., 2015	Italy	excluded, (5)
Found in a review	1	23884951	Anodal transcranial direct current stimulation temporarily reverses age-associated cognitive decline and functional brain activity changes	Meinzer et al., 2013	Germany	included
Found in a previously listed paper	1	25346688	A double-blind randomized clinical trial on the efficacy of cortical direct current stimulation for the treatment of Alzheimer's disease	Khedr et al., 2014	Egypt	included
Transcranial direct current stimulation, memory	194	27555381	Effects of anodal transcranial direct current stimulation and serotonergic enhancement on memory performance in young and older adults	Prehn et al., 2017	Germany	included
Manenti, Sandrini	4	29259554	Strengthening of existing episodic memories through non-invasive stimulation of prefrontal cortex in older adults with subjective memory complaints	Manenti et al., 2017	Italy	included
Ferrucci	41	18525028	Transcranial direct current stimulation improves recognition memory in Alzheimer's disease	Ferrucci et al., 2008	Italy	included
		16843494	Effects of transcranial direct current stimulation on working memory in patients with Parkinson's disease	Boggio et al., 2006	Brazil	excluded, (2)
		21840288	Prolonged visual memory enhancement after direct current stimulation in Alzheimer's disease	Boggio et al., 2012	Italy, Brazil	included
Berryhill, Jones	9	22684095	tDCS selectively improves working memory in older adults with more education	Berryhill and Jones, 2012	USA	excluded, (2)
PubMed studies (that will be added to tDCS database)	4	29736192	The effects of transcranial direct current stimulation on the cognitive functions in older adults with mild cognitive impairment: A pilot study	Cruz Gonzalez et al., 2018	Hong Kong	included
		30395314	Effects of transcranial direct current stimulation on episodic memory in amnestic mild cognitive impairment: A pilot study	Manenti et al., 2020	Italy or UK	excluded, (6)
		29313802	Augmenting cognitive training in older adults (The ACT Study): Design and Methods of a Phase III tDCS and cognitive training trial	Woods et al., 2018	USA	excluded, (4)
		30783198	tDCS-induced episodic memory enhancement and its association with functional network coupling in older adults	Antonenko et al., 2019	Germany	excluded, (2)

* Results tabulated include studies prior to application of inclusion/exclusion criteria with indication whether the study was included or excluded as well as reasons for exclusion. *Reasons for exclusion: (1) cognitive (declarative or working memory) outcome measures of tDCS effects are not a focus of the study, (2) study design insufficient (single-blinded, not sham/placebo controlled), (3) blinding procedure not recorded, (4) publication only contains study protocol, (5) case study, (6) restricted access to the paper until submission of this review, (7) age criterion not fulfilled.

**Table 2:** Summary of all studies reviewed including most important features and tDCS parameters.

**References (PMID)**	**Study design**	**Participants** (N, female/male, age [mean ± SD and/or range], condition^*^)	**Drop-outs**	**Stimulation parameters**					**Behavioral (cognitive) effects**
				**Montage**	**Intensity**	**Duration**	**# Active tDCS sessions**	**Timepoint of tDCS**	
Im et al. (2019) (31196835)	Sham-controlled, double-blinded, randomized	*N* = 18, 15/3, 73.4, 60–85, early AD	2	Anode F3, cathode F4	2 mA	30 min	Every day for 6 months	Baseline	+
Sandrini et al. (2016) (26923418)	Sham-controlled, double-blinded, randomized	*N* = 28, 17/11, 68.9, healthy	None	Anode F3, cathode right supraorbital region	1.5 mA	15 min	Up to 5	During learning phase	+
Meinzer et al. (2015) (25449530)	Sham-controlled, double-blinded, randomized, counterbalanced	*N* = 36, 14/22, 69.56 ± 5.56 (healthy group), 67.44 ± 7.27 (MCI group), healthy and MCI	None	Anode left ventral IFG, cathode right supraorbital area	1 mA	20 min	1	During rs- and task-related fMRI (semantic word retrieval)	+
Yun et al. (2016) (27903289)	Sham-controlled, double-blinded, randomized	*N* = 16, 11/5, 73.9, 65–86, MCI	None	Anode F3, cathode F4	2 mA	30 min	9 (in 3 weeks)	Baseline	+
Fiori et al. (2017) (28062255)	Sham-controlled, double-blinded, randomized, counterbalanced	*N* = 30, 29 ± 6 20–40 (young group), 72 ± 6 60–80 (old group), healthy	None	Unihemispheric: anode CP5, cathode right orbito-frontal cortex; bihemispheric: anode CP5 cathode CP4	2 mA	20 min	2 (uni- and bi-hemispheric)	During retrieval phase	+
Westerberg et al. (2015) (26116933)	Sham-controlled, double-blinded, randomized	*N* = 18, 16/3, 73.4 65–85, healthy	None	Anodes F7 and F8, references to ipsilateral mastoids	so-tDCS: 0.75 Hz, 0-260 μA	5 times 5 min	1	During sleep	+
Cotelli et al. (2014) (24678298)	Sham-controlled, double-blinded, randomized	*N* = 36, 29/7, 76.5, probable mild to moderate AD	2 before 3-months, 4 before 6-months follow-up	Anode left DLPFC (8 cm frontally, 6 cm laterally), cathode right deltoid muscle	2 mA	25 min	10 (in 2 weeks)	During memory or motor training	−
Bystad et al. (2016) (27005937)	Sham-controlled, double-blinded, randomized	*N* = 25, 14/11, 72.5 (AD group); *N* = 22, 18/4, 68.8 ± 6.8, 59–83 (healthy group), AD and healthy	None	Anode T3, cathode FP2	2 mA	30 min	6 (in 10 days)	Baseline	−
Lu et al. (2019) (31529691)	Sham-controlled, double-blinded, randomized	*N* = 173, 108/65, 74, 60–90, NCD-AD	28	Anode T3, cathode contralateral upper limb	2 mA	20 min	12 (in 3 weeks)	During WM training	+
Meinzer et al. (2013) (23884951)	Sham-controlled, double-blinded, within-subject	*N* = 20, 10/10, 26.4 ± 3.4 19–31 (young group), 68 ± 5.7 60–76 (old group), healthy	None	Anode left ventral IFG, cathode right supraorbital area	1 mA	20 min	1	During rs- and task-related fMRI (semantic word retrieval)	+
Khedr et al. (2014) (25346688)	Sham-controlled, double-blinded, randomized	*N* = 34, 15/19, 69.7 ± 4.8 62–79, mild to moderate AD	None	atDCS: anode LDLPFC, cathode contralateral supraorbital region; ctDCS: vice versa	2 mA	25 min	10 consecutive days	Baseline	+
Prehn et al. (2017) (27555381)	Sham-controlled, double-blinded, randomized	*N* = 39, 23/17, 24 ± 4 18–35 (young group), 66 ± 7 50–80 (old group), healthy	1	Anode T6, cathode contralateral frontopolar cortex	1 mA	20 min	2	During learning phase	+
Manenti et al. (2017) (2925955)	Sham-controlled, double-blinded, randomized	*N* = 22, 14/8, 74.5 ± 5.9, SMC	None	Anode F3, cathode right supraorbital area	1.5 mA	15 min	1	After learning phase but before recall	+
Ferrucci et al. (2008) (18525028)	Sham-controlled, double-blinded, randomized, cross-balanced	*N* = 10, 7/3, 75.2 ± 7.3 64–84, probable AD	None	Anode P3-T5 left and P6-T4 right, cathode contralateral deltoid muscle	1.5 mA	15 min	2 (anodal and cathodal)	Between tasks	+
Boggio et al. (2012) (21840288)	Sham-controlled, double-blinded, randomized, counterbalanced	*N* = 15, 7/8, 77.5 ± 6.9 (Italian group), 80.6 ± 9.5 (Brazilian group), AD	None	Anodes bilaterally T3 and T4, cathode right deltoid muscle	2 mA	30 min	5 consecutive days	Baseline	+
Cruz Gonzalez et al. (2018) (29736192)	Sham-controlled, single-subject study A-B-C-A design	*N* = 5, 2/3, 72.8 ± 6.6, 67–81, MCI	1 before last baseline session	Anode F3, cathode contralateral deltoid muscle	2 mA	30 min	1–5 (in 1 week)	During cognitive training	+

*Disease conditions:*

* *AD, Alzheimer's Disease; SCD, subjective cognitive decline; MCI, mild cognitive impairment; NCD-AD, neurocognitive disorder due to Alzheimer's Disease; SMC, subjective memory complaints*.

## Results

### Overview

The 16 studies that met all inclusion criteria were performed between 2008 and 2019 (more recent publications had to be excluded, see Table 1) and included 543 subjects comprising 60.8% females and 39.2% males. Thirty-eight subjects dropped out during the course of the respective study making a total drop-out rate of 7.1%. All older participants were aged between 50 and 90 years (only 2 studies included younger control groups). Five out of 16 studies included only healthy elderlies (Meinzer et al., 2013; Westerberg et al., 2015; Sandrini et al., 2016; Fiori et al., 2017; Prehn et al., 2017), while the remaining studies applied tDCS to patients suffering from MCI, subjective memory decline (SMC), neurocognitive disorder due to AD (NCD-AD) or probable as well as mild to moderate AD (Ferrucci et al., 2008; Boggio et al., 2012; Cotelli et al., 2014; Khedr et al., 2014; Meinzer et al., 2015; Bystad et al., 2016; Yun et al., 2016; Manenti et al., 2017; Cruz Gonzalez et al., 2018; Im et al., 2019; Lu et al., 2019). In order to evaluate the effectiveness of tDCS protocols applied to patients suffering from different age-associated diseases, the results section considers outcomes in healthy subjects and patients with the above listed cognitive diseases separately. Thereby, we aim to provide an overview of limitations and successes of tDCS in patients in comparison to healthy individuals. We think that efficient stimulation methodologies to treat age-related cognitive decline can only be proposed when considering disease-related variability in tDCS efficiency. Variability may exist when comparing applications in healthy vs. diseased brains but also in the different age-associated diseases as well as different disease states due to varying degrees of progression of neurodegeneration or different brain areas affected.

### TDCS in Healthy Elderly People

To begin with, assuming that tDCS has the potential to modulate cognitive functions in healthy aging, Meinzer et al. combined anodal tDCS during an overt semantic learning task with functional magnetic resonance imaging (fMRI) to investigate effects on task performance as well as local brain activity. The main outcome of this study was enhanced word retrieval and restoring of “youth-like” network connectivity in old subjects after receiving unihemispheric anodal tDCS to the left ventral inferior frontal gyrus in comparison to the old and young sham groups (Meinzer et al., 2013). Based on this, Fiori et al. tried to assess whether bihemispheric tDCS over temporo-parietal areas (with the anode on the left and the cathode on the right contralateral hemisphere) differently impacts the performance in a verbal learning task in old vs. young subjects in comparison to unihemispheric tDCS. Here, stimulation did not affect the performance in young participants while older subjects seemed to profit from bihemispheric tDCS manifested in significantly higher numbers of correctly retrieved words (Fiori et al., 2017). Both studies referred to evidence on age-related altered network connectivity and aimed to compensate for “bihemispehric hyperactivities.” Another study investigated combined effects of tDCS and selective serotonin reuptake inhibitors (SSRIs) on healthy cognition in elderly people (Prehn et al., 2017). Prehn et al. assumed that this combination of two potential cognition-enhancing methodologies might lead to synergistic effects and thus ameliorate memory performance. The assessment of object-location learning indicated that a combination of SSRIs and tDCS but not single-modality treatment improved immediate memory but surprisingly worsened learning performance in comparison to other conditions. However, this was one of the only studies placing the anode on the right (temporal) cortex (Prehn et al., 2017). Sandrini et al. showed that anodal tDCS over the left dorso-lateral prefrontal cortex (LDLPFC) improved delayed recall in comparison to sham tDCS in old subjects after a verbal episodic memory task when applied during the learning phase (Sandrini et al., 2016). Finally, Westerberg et al. applied bilateral anodal sinusoidal slow-oscillatory tDCS (so-tDCS) with a frequency of 0.75 Hz to the mid-lateral frontal cortex of healthy elderlies during sleep, hypothesizing that age-related memory decline could be a consequence of decreased memory consolidation during altered sleep upon aging. So-tDCS enhanced verbal recall in old participants in comparison to sham so-tDCS and slow-oscillatory activity in the frontal lobe (Westerberg et al., 2015).

### TDCS in Age-Associated Diseases

#### Mild Cognitive Impairment and Subjective Memory Complaints

Expanding their examinations on the potential of tDCS to counteract cognitive decline, Meinzer et al. performed another study applying a similar tDCS and fMRI methodologies as in Meinzer et al. (2013) to patients with MCI (Meinzer et al., 2015). In baseline conditions patients performed significantly worse in a word retrieval task compared to elderly healthy controls. However, word-retrieval performance was significantly ameliorated up to the level of controls after anodal tDCS over the left ventral IFG (Meinzer et al., 2015). Yun et al. found that repeated application of anodal tDCS over the DLPFC (nine times 30 min in 3 weeks) significantly increased brain metabolism in MCI patients (measured by FDG-PET) and enhanced memory performance compared to sham tDCS (Yun et al., 2016). Anodal tDCS applied over the left lateral PFC after learning and before recall of an episodic memory task in patients with subjective memory complaints (SMC) significantly increased word recognition performance up 30 days after learning in comparison to the sham group (Manenti et al., 2017). Moreover, in a pilot study of Cruz Gonzalez et al. anodal or cathodal tDCS over the DLPFC was combined with cognitive training during stimulation to synergistically enhance declined cognition in MCI. Tendencies of increased processing speed, selective attention, working memory activities, and the completion time in planning ability and divided attention tasks were observed for both anodal and cathodal stimulation in comparison to sham tDCS. However, due to the small sample size and the lack of randomization, results were highly variable and need further investigation and confirmation (Cruz Gonzalez et al., 2018). The biggest study (including 201 participants) has been performed by Lu et al. who also combined tDCS over left temporal areas and (working) memory training in patients with neurocognitive disorder due to AD (NCD-AD). Participants underwent 12 sessions of anodal tDCS in 3 weeks and performed working memory tasks during stimulation. Performance significantly increased up to 8 or even 12 weeks post-intervention in secondary outcome measures (delayed recall, working memory tests, logical memory) for subjects receiving tDCS and working memory training compared to control groups. However, primary outcomes (global cognition measured by ADAS-Cog and the working memory training performance) improved throughout all groups without stimulation-dependent differences (Lu et al., 2019).

#### Alzheimer's Disease

Two of the first small studies to investigate tDCS in patients with AD were performed by Ferrucci et al. in 2008 and Boggio et al. in 2012. Ferrucci et al. applied anodal and cathodal tDCS to the temporo-parietal cortex and were able to show that a single session of anodal tDCS significantly increased accuracy in a word recognition task while cathodal tDCS had contrary effects. However, no stimulation-type-dependent changes in reaction times were found based on the assessment of a visual attention task (Ferrucci et al., 2008). Subsequently, Boggio et al. used bilateral anodal tDCS applied for five consecutive days over the temporal cortex, which significantly ameliorated performance of AD patients in a visual recognition task but not in a visual attention task compared to sham tDCS (Boggio et al., 2012). Examining longer-term effects of 10 sessions of anodal tDCS over the LDLPFC on cognitive abilities in AD, Khedr et al. found that MMSE scores significantly improved for both anodal and cathodal stimulation compared to sham tDCS even 2 months post-intervention (Khedr et al., 2014). Cotelli et al. also applied 10 sessions of tDCS over the LDLPFC in AD patients but combined with individualized memory training during stimulation. This study failed to show a significant effect of anodal tDCS on memory performance in AD (Cotelli et al., 2014). Similarly, Bystad et al. could not reveal significant effects of anodal tDCS applied over the left temporal cortex in subjects suffering from AD. Verbal memory test scores did not differ significantly after active stimulation in comparison to sham. However, a tendency of increased delayed recall was observed for the group receiving active tDCS (Bystad et al., 2016). Finally, the findings of Im et al., who studied the effects of 6-months daily at home tDCS in AD patients, were in line with Khedr et al. (2014). The main outcomes were significant benefits of anodal tDCS on global cognition assessed via MMSE and improved language function based on ameliorated performance in the Boston Naming Test (BNT), stabilization of some executive functions in AD patients compared to patients receiving sham stimulation as well as increased cerebral glucose metabolism (Im et al., 2019).

## Discussion

### Methodological Considerations

**Figure 2:**
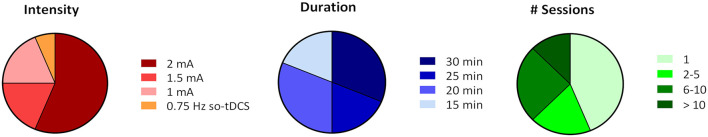
Relative comparison of tDCS parameters intensity, session duration, and session number (# session) chosen in the 16 reviewed studies. Percentages were calculated based on the number of studies that chose a certain parameter out of the total number of 16 studies and do not resemble relative frequencies based on the number of subjects.

In the 16 reviewed studies tDCS intensity varied between 1 and 2 mA [except for the study of Westerberg et al. (2015) who applied so-tDCS with a frequency of 0.75 Hz and 0–260 μA intensity], one session lasted between 15 and 30 min and for most studies the number of sessions varied between 1 and 10 (Figure 2). Exceptions in session number were the study of Lu et al. (2019) who applied 12 sessions of tDCS and Im et al. (2019) who chose to use daily at home tDCS over 6 months to treat patients with AD (Figure 2).

In the majority of applications stimulation intensity was rather high (2 mA) and most of the sessions lasted 25–30 min. Importantly, none of the studies reported severe adverse effects resulting from tDCS or so-tDCS. In 3 studies (Khedr et al., 2014; Sandrini et al., 2016; Lu et al., 2019) rarely occurring mild side effects were skin irritation, itching, and redness under the area of the electrodes. In only 2 studies (Prehn et al., 2017; Cruz Gonzalez et al., 2018) a few subjects reported a mild headache and dizziness after the stimulation, which only lasted for several hours. However, the occurrence of mild adverse effects did not seem to correlate with the magnitude of stimulation intensity, session duration or session number.

**Figure 3:**
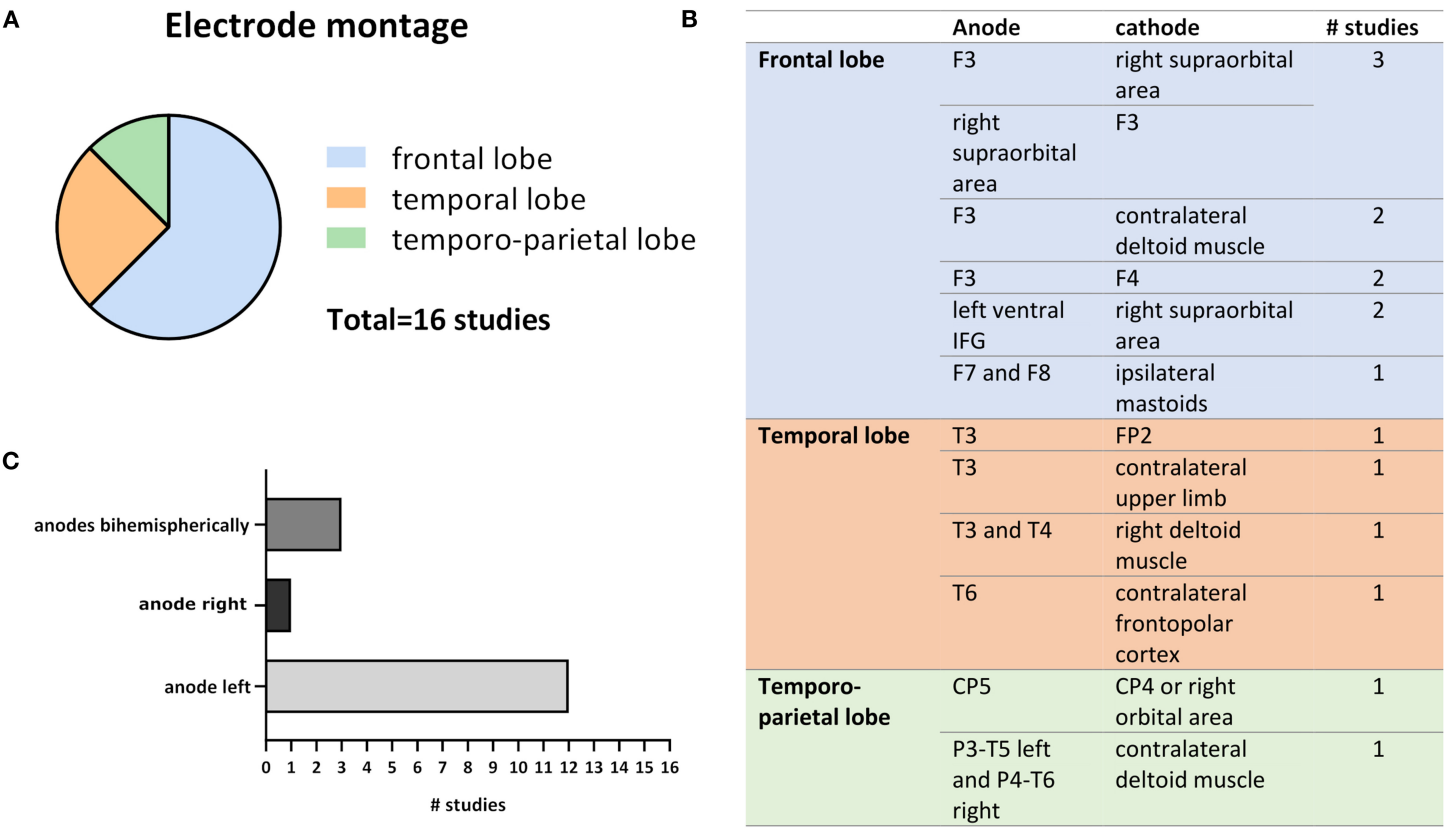
Variations in electrode montages extracted from the 16 reviewed studies. **(A)** Shows the relative numbers of studies out of all 16 studies that chose frontal, temporal or temporo-parietal cortical targets for tDSC. **(B)** Depicts exact anode and cathode positions as well as the number of studies that applied respective montage. **(C)** Compares the frequency of anodal left, right, and bihemispheric cortical stimulation (the anode is usually considered as the active electrode).

A more precise investigation of electrode montage revealed that 12 out of 16 studies stimulated the left cortical hemisphere, mostly targeting the (pre-)frontal cortex (Figure 3). However, several studies also stimulated temporal or temporo-parietal areas (Figure 3). The difference in stimulation location may be traced back to deviating hypotheses and different aims in modulating cognitive functions. All but 1 study, that targeted the temporal or temporo-parietal cortex, aimed to ameliorate or slow AD progression, as the medial temporal lobe (MTL), including the hippocampus, is one of the major and earliest affected brain regions in disease (Smith, 2002; Dickerson et al., 2004). The reason for targeting the temporal cortices might be to reach areas that are mainly affected by decline of neuroplasticity due to neurodegeneration and thereby potentially counteract the loss of neuronal connections. Although episodic memory is thought to mainly depend on intact functioning of MTL and hippocampus (Dickerson and Eichenbaum, 2010), the PFC and non-disturbed communication between all these areas seem crucial in cognitive processes relying on episodic memory (Fletcher and Henson, 2001; Brem et al., 2013).

Scientists targeting the frontal lobe (mainly the DLPFC) mostly aimed to modify cognitive processes by directly impacting underlying neuronal networks and indirectly subcortical areas (Frith and Dolan, 1996). Of the two studies that failed to show significant effects of tDCS on cognitive functions Cotelli et al. (2014) targeted the LDLPFC, while Bystad et al. (2016) stimulated the left temporal cortex. In both studies the subjects were suffering from AD. Because neuronal network connectivity and synchrony seem to change upon aging (Goh, 2011; Meinzer et al., 2013), further or increased alterations might occur in disease, which should be considered when developing new tDCS protocols to treat patients with cognitive impairment or advanced dementia. It might be beneficial to include individual computational modeling of current distribution to account for structural brain alterations happening upon aging such as atrophy along with raising volumes of the ventricular system (Fjell and Walhovd, 2010). Indeed, increased cerebrospinal fluid (CSF) volume and reduced tissue density significantly impact current distribution throughout the brain (Opitz et al., 2015) as conductivity is higher in more aqueous media and tissues. This was further confirmed in a recent study by Antonenko et al. who used computational modeling to show that the cerebral electric field induced by transcranial electrical stimulation is higher for young compared to older people (Antonenko et al., 2021). In the studies reviewed here, mean age of participants differs up to 13 years (Prehn et al., 2017: 66 years; Boggio et al., 2012: 79.1 years) (Table 2) which exemplifies that age differences also occur in studies of the elderly. Age-related electrical field variations may cause controversial results, even when comparing studies performed within similar age groups but with significant mean age deviations. In addition to age-related increase of brain atrophy, individual head anatomy seems to impact the induced electric field strengths (Antonenko et al., 2021). Computational models have shown that large electrodes which are most frequently used in tDCS studies produce large diffuse electric fields in the brain. Not only strengths but also the distributions of these fields are highly dependent on individual head and brain anatomy. Lately, smaller electrodes as well as novel montages, including high-definition tDCS (HD-tDCS) arrangements have been introduced to improve the focality of the stimulation. However, a recent study just reported that better electric field focality was achieved only at the cost of increased interindividual variability (Mikkonen et al., 2020). Nevertheless, another recent study using HD-tDCS and current modeling demonstrated that focal current delivery to the DLPFC with sufficient magnitude of the induced current, modulated the neural activity in older adults (Gbadeyan et al., 2019). However, it remains to be elucidated whether more precise stimulation localization is beneficial in patients suffering from cognitive decline that is mostly caused by neurodegeneration in multiple brain areas affecting widespread cortical networks rather than precisely localized brain regions. Altogether, this highlights the importance of individually predicting the electric field distribution by means of structural brain imaging combined with computational modeling as this may be a crucial factor when applying tDCS to aging brains and lead to decreased effect variability as well as ameliorated spatial accuracy.

The outcomes of the reviewed studies show a high degree of variability—in the results themselves but also in their respective measures (Table 3). Consequently, to reduce variability, the application of multiple and precise cognitive outcome measures that assess a representative range of cognitive functions, is essential when performing tDCS experiments that aim to modulate cognition in the elderly. It seems like the effect of tDCS can sometimes be rather specific for single aspects of human cognition. This might correlate with the part of the cortex that has been stimulated, however, it needs to be pointed out that the spatial resolution of tDCS is rather low. The use of screening tools such as widely applied MMSE or MoCA to evaluate effects on global cognition may be insufficient as these tests resemble a very limited spectrum of cognitive functions and have been developed for quick clinical diagnoses and screenings. Moreover, only few of the reviewed studies included both physiological and cognitive measures. The combination of extensive standardized cognitive assessments with physiological methods such as EEG or fMRI may reveal origins of variability and facilitate the evaluation of tDCS effects.

**Table 3:** Summary of all studies reviewed, listing respective cognitive assessments including the timepoints of the assessment, exact measures, and main outcomes.

**References**	**Cognitive assessment (to evaluate tDCS effects on cognition)**		
	**Timepoints**	Measures^*^	**Outcomes**
Im et al. (2019)	Baseline and after 6 months of treatment	MMSE, CDR, neurological test battery (digit span test, BNT, RCFT with immediate and delayed recall and recognition, clock drawing test, SVLT with immediate and delayed recall and recognition, contrasting program, Go-no go test, COWAT, Stroop word and color reading)	• MMSE and BNT scores significantly improved after active tDCS compared to sham • Active tDCS resulted in consistent performance (at lower score levels) in contrasting program and Stroop word reading while scores decreased for sham
Sandrini et al. (2016)	Learning performance, recall after 48 h and recall after 1 month	Learning and recall of a list of 20 words	• Significant effect for recall after 48 h: Active tDCS group recalled significantly more words compared to sham • No significant differences after 1 month
Meinzer et al. (2015)	During stimulation (and fMRI)	Semantic word retrieval task	• tDCS significantly improved semantic word-retrieval performance in the patients to the level of controls
Yun et al. (2016)	Baseline and after 3-weeks of treatment	MMQ (MMQ-C, MMQ-A, MMQ-S)	• MMQ-C significantly increased after active tDCS compared to sham • Results for MMQ-A were similar to MMQ-C results but not significant between the active and sham groups • No significant difference for MMQ-S between active tDCS and sham
Fiori et al. (2017)	During word retrieval: 1–10 presentations for each picture-word pair (T1-T10)	Training, verification, and word retrieval of 20 pseudoword-picture associations (bisyllabic pseudowords)	• Bihemispheric: higher number of correct responses in the old group during T10 compared to T1 compared to unihemispheric and sham condition • No differences between the three conditions in the young age group • During T10 the young group was significantly more accurate than the old group for unihemispheric and sham; no significant difference in the bihemispehric condition • Same results for vocal reaction times
Westerberg et al. (2015)	Before a 90-min nap and 30 min after	Two declarative memory tests (word-pair recall, fast recognition test), 1 non-declarative test (object-priming test)	• Recall improvement from pre-nap to post-nap was significantly larger for active so-tDCS compared to sham • No significant fast recognition or object priming performance difference between active and sham so-tDCS after the nap (both increased significantly)
Cotelli et al. (2014)	Baseline (T0), after 2 weeks of treatment (T1), after 3 months (T2), after 6 months (T3)	Face-name association task (FNAT), neuropsychological tests (picture naming task, BADA, Rivermead behavioral memory test, Rey auditory verbal learning test, Rey-Osterrieth test, complex figure copy, TMT A and B)	• FNAT: active or sham tDCS + memory training group showed significantly improved performances compared to active tDCS + motor training group at T1 and similar for T2, at T3 sham + memory training was still significant compared to the other two groups • No differences in neuropsychological tests (except an improvement for both tDCS and sham + memory training at T3 in the TMT A score)
Bystad et al. (2016)	Before and after stimulation	Primary: immediate and delayed recall and recognition of CVLT-II Secondary: MMSE, clock drawing test, TMT A and B	• CVLT-II: no significant differences between active and sham tDCS but a tendency toward higher improvement in CVLT-II recall after active tDCS • No significant differences for secondary outcome measures
Lu et al. (2019)	Baseline (T0), after 4 weeks of treatment (T1), 4 weeks after post-intervention (T2), 8 weeks after post-intervention (T3)	Primary: WM test (RT), ADAS-Cog Secondary: CVFT, TMT, Chinese neuropsychiatric inventory (CNIP)	• ADAS-Cog: significant improvement for all groups at T1, but no difference between groups, tendency of falling back to baseline at T2 and T3 for all groups • WM test: significant improvement for all groups until T3, tDCS+WMT showed highest WM capacity at T1 compared to other groups • CVFT: tDCS-WMT showed a greater improvement in delayed recall compared to single-modality interventions; at T3 only the tDCS+WMT group showed significant enhancement on delayed recall performance over baseline • tDCS-WMT group showed better performance of logical memory at 12th week
Meinzer et al. (2013)	During stimulation (and fMRI)	Overt semantic word generation task	• During sham younger adults produced significantly less errors than elderly • Older subjects produced significantly less errors during active tDCS in comparison to sham
			• Response times (RTs) were comparable between young and old subjects during sham; no difference in RTs for elderly during active tDCS compared to sham
Khedr et al. (2014)	Baseline (T0), after 10 days of treatment (T1), after 1 month (T2), after 2 months (T3)	MMSE, WAIS-III (verbal comprehension, arithmetic and digit span, perceptual organization, processing speed)	• MMSE: significant improvement in anodal and cathodal tDCS compared to sham (increase of nearly 2 points at T1 and further increase of 2 points at T2 and T3); anodal tDCS group showed better improvement in orientation, registration, attention, and naming object compared to cathodal tDCS • WAIS-III: only cathodal and not anodal tDCS showed improved performance IQ compared to sham
Prehn et al. (2017)	Immediate recall, delayed recall after 6 h, 1 day later and 1 week later	Object-location learning task (LOCATO), primary outcome: immediate recall, secondary outcome: delayed recall	• Significant effect of SSRI but not of stimulation on immediate recall scores • Young and old group profited most from atDCS+SSRI • No significant effects on delayed recall
Manenti et al. (2017)	Baseline (after learning), free recall and recognition 48 h and 30 days after learning (and tDCS)	Learning, recall, and recognition of a list of 20 words	• Significant difference on hit-false alarms score between atDCS and sham at day 30, anodal tDCS significantly improved memory recognition on day 30 • atDCS and sham group showed similar free recall performance at day 30
Ferrucci et al. (2008)	Baseline and 30 min after stimulation	Word recognition task (WRT), visual attention task	• atDCS improved WRT accuracy, while ctDCS significantly worsened it, sham left it unchanged; same results for DI (derived by subtracting false positive from true positive responses) • No significant differences in RTs in the visual attention task for atDCS or ctDCS compared to sham
Boggio et al. (2012)	Baseline (T0), at the end of treatment day 5 (T1), 1 week later (T2), 4 weeks later (T3)	MMSE, ADAS-Cog, visual recognition task (VRT), visual attention task (VAT)	• No significant effects for MMSE, ADAS-Cog, and VAT scores between active and sham tDCS • VRT: significant main effect for tDCS performance changes from baseline: 8.99% after anodal and 2.62% after sham tDCS (for T1, T2 and T3)
Cruz Gonzalez et al. (2018)	Screening, baseline (after CS training), after sham+CS, after tDCS+CS, post assessment (after CS)	Cognitive stimulation (planning ability and divided attention, processing speed and selective attention, short-term memory, calculation and WM), CDR, MoCA	• Enhanced cognitive performance in processing speed, selective attention, WM activities, completion time in planning ability and divided attention tasks for active tDCS compared to sham • Variable CS outcomes but subjects did not show significantly better outcomes in sham intervention compared to baseline CS

*The only two studies that did not reveal significant effects are highlighted in gray. Cognitive measures*:

* *MMSE, Minimal Mental State Examination; CDR, Clinical Dementia Rating (Morris, 1993); BNT, Boston Naming Test; RCFT, Rey Complex Figure Test; SVLT, Seoul Verbal Learning Test; COWAT, Controlled Oral Word Association Test; MMQ, Multifactorial Memory Questionnaire; BADA, Battery for the Analysis of the Aphasic Deficit; TMT, Trail Making Test; CVLT-II, California verbal learning test second edition; WM, Working Memory; ADAS-Cog, Alzheimer's Disease assessment scale-cognition subscale; CVFT, Category Verbal Fluency Test; WAIS-III, Wechsler Adult Intelligence Subscales; MoCA, Montreal Cognitive Assessment; CS, Cognitive Stimulation*.

Out of all studies only three combined tDCS with cognitive training (Cotelli et al., 2014; Cruz Gonzalez et al., 2018; Lu et al., 2019). Even though results depicted here are not very consistent, the idea of synergistic amelioration and intervention of cognitive decline, by combining methods that positively impact cognitive functions in the elderly, seems promising. However, when assessing the effects as well as comparing active to sham stimulation conditions it needs to be considered that cognitive training itself might already improve cognition in both groups. Consequently, effects of tDCS may result in only slight differences that might be hard to detect using semi-sensitive cognitive outcome measures. Further some participants might not respond to the stimulation. A relatively high number of participants is important to properly assess the effects of tDCS on cognition of elderly people. Therefore, future studies may be designed in a multicentric fashion to increase participant numbers and thus reliability of experimental outcomes.

When treating diseases such as MCI or AD, it is crucial to consider long-term (LT) effects of tDCS. We define LT effects as those measured at least 1 week after the end of the intervention. Among the studies reviewed here, only seven examined LT effects (Boggio et al., 2012; Cotelli et al., 2014; Khedr et al., 2014; Sandrini et al., 2016; Manenti et al., 2017; Prehn et al., 2017; Lu et al., 2019) (Table 3), of which only 5 revealed significant results (Boggio et al., 2012; Cotelli et al., 2014; Khedr et al., 2014; Manenti et al., 2017; Lu et al., 2019) meaning that at least one cognitive test score was significantly better at LT timepoints (after stimulation) compared to either baseline (before stimulation) or to the respective control condition (e.g., sham stimulation). When comparing active vs. sham stimulation, only three out of these five studies revealed significant improvement of the active group over the sham group at LT timepoints (Boggio et al., 2012; Khedr et al., 2014; Manenti et al., 2017). Interestingly, the two studies that did not find significant LT effects when comparing active to sham stimulation used a combination therapy of tDCS and cognitive training (Cotelli et al., 2014; Lu et al., 2019). In both studies the tDCS sham group received cognitive training. Taken together, this indicates that, as suggested above, both methods—tDCS and cognitive training—can positively impact cognition in the elderly and both potentially result in LT effects. Whether a combination of both methods enhances LT effects remains to be elucidated. A possible explanation for the absence of LT effects in the remaining two studies (Sandrini et al., 2016; Prehn et al., 2017) is that the total time of stimulation (duration of one session multiplied by the number of sessions) applied by Sandrini et al. (15–75 min; stimulation time varied between subjects as stimulation was repeated until a certain test score was achieved) and Prehn et al. (40 min) deviates strongly from the mean time of stimulation (181 min) of all studies that showed significant LT effects.

### Future Perspectives

In conclusion, based on recently available data (http://tdcsdatabase.com) to counteract age-associated cognitive decline, anodal tDCS should be applied repeatedly to the left cortical hemisphere. In adulthood, several cognitive processes show dominant activity in the left cortex, while cognitive decline upon aging seems to correlate with network alterations and “bihemispheric hyperactivity” (Goh, 2011; Antonenko et al., 2012; Meinzer et al., 2013). Targeting the LDLPFC may be one of the most effective possibilities as human cognition highly depends on cortical as well as subcortical networks involving the PFC (Frith and Dolan, 1996). Further, tDCS has very little mild adverse effects, which seem to depend on subjective sensation rather than stimulation parameters, so that a stimulation intensity of 2 mA may be chosen and sessions could last up to 30 min without risking significant side effects. Moreover, LT effects should be considered in future studies as they are advantageous for therapy considering the following aspects. Even though stimulators are now small and mobile, and the stimulations could be performed regularly by patients themselves after being trained by a specialist, at home tDCS is time consuming and requires certain skills as well as mobility. Independent application is particularly difficult for patients with cognitive disorders such as MCI or AD and a trained assistant such as a relative or a family doctor would be required to perform the stimulations properly. Additionally, in some cases repetitive stimulation may cause minor side effects as described above. Considering these limitations, treatment effects should outlast the time during stimulation, especially for application of tDCS in elderly people with cognitive impairment, and likewise persist in LT measurements.

In the field of NIBS research, stimulation interventions have so far mostly focused on group-based, general protocols. While standardization of study protocols may increase comparability which potentially facilitates translation of experimental studies into clinical applications, it can also be a major limitation of this methodology. Generalized stimulation practices might miss to fully consider the underlying mechanisms in the individual brain that guide the effective response to a given intervention. Therefore, NIBS protocols leveraging on the combination of stimulation approaches with electrical field modeling, neuroimaging and electrophysiology (Esmaeilpour et al., 2020) could advance the characterization of personalized response and prognostic biomarker discovery. This will result in a better understanding and reduction of variability of the response to stimulation. However, simulations of individual brains cannot be perfect due to uncertainties of the model parameters (e.g., conductivity) and EEG as well as fMRI methods both suffer from electric field artifacts. A recent study suggests the functional near-infrared spectroscopy (fNIRS) may be a better neuroimaging technique in order to study the hemodynamics response evoked by tDCS and consequently better dosing the stimulation (Arora et al., 2021). Indeed, a recent study investigated the feasibility of portable neuroimaging of cerebellar tDCS in conjunction with electroencephalography (EEG) to measure changes in the brain activation at the PFC and the sensorimotor cortex (SMC) in hemiparetic chronic stroke survivors. It was observed that there is a clear relationship between mean lobular electric field strength and oxy-hemoglobin concentrations/log10-transformed EEG band power. Nevertheless, future studies are needed to investigate and replicate these effects in a larger cohort and to clearly discriminate non-responders from responders. Afterall, an extended meta-analysis of the here reviewed studies and respective results could contribute to further specification and suggestions for future tDCS studies aiming to introduce novel treatment approaches to intervene with age-related cognitive deterioration as well as neurodegeneration.

## Data Availability Statement

The original contributions presented in the study are included in the article/supplementary material, further inquiries can be directed to the corresponding author.

## Author Contributions

AS performed database research and analysis, prepared figures and tables, and wrote the manuscript. LD reviewed and contributed to the manuscript. AA supervised the database research and analysis and reviewed the manuscript. All authors contributed to the article and approved the submitted version.

## Funding

This study was supported by the Ministry of Lower Saxony for Science and Culture (76251-12-7/19; ZN 3456). We acknowledge support by the German Research Foundation and the Open Access Publication Funds of the Georg–August University Göttingen, Germany.

## Conflict of Interest

The authors declare that the research was conducted in the absence of any commercial or financial relationships that could be construed as a potential conflict of interest.

## Publisher's Note

All claims expressed in this article are solely those of the authors and do not necessarily represent those of their affiliated organizations, or those of the publisher, the editors and the reviewers. Any product that may be evaluated in this article, or claim that may be made by its manufacturer, is not guaranteed or endorsed by the publisher.
